# Low risk for transmission of zoonotic *Giardia duodenalis* from dogs to humans in rural Cambodia

**DOI:** 10.1186/1756-3305-7-412

**Published:** 2014-08-29

**Authors:** Tawin Inpankaew, Fabian Schär, Peter Odermatt, Anders Dalsgaard, Wissanuwat Chimnoi, Virak Khieu, Sinuon Muth, Rebecca J Traub

**Affiliations:** Department of Veterinary Disease Biology, Faculty of Health and Medical Science, University of Copenhagen, Copenhagen, Denmark; Department of Parasitology, Faculty of Veterinary Medicine, Kasetsart University, Bangkok, Thailand; Swiss Tropical and Public Health Institute, Basel, Switzerland; University of Basel, Basel, Switzerland; National Center for Parasitology, Entomology and Malaria Control, Phnom Penh, Cambodia; Faculty of Veterinary and Agricultural Sciences, The University of Melbourne, Parkville, Victoria Australia

**Keywords:** *Giardia duodenalis*, Humans, Dogs, Zoonosis, Cambodia

## Abstract

**Background:**

A number of epidemiological studies have demonstrated *Giardia* as prevalent in both humans and dogs worldwide and have postulated the occurrence of anthroponotic, zoonotic and animal-specific cycles of transmission, which may be geographically and regionally unique in its epidemiology. The aim of this study was to utilise molecular tools to determine the prevalence and compare genotypes of *Giardia duodenalis* infecting humans and dogs living in a previously identified *Giardia*-endemic village in rural Cambodia in order to ascertain zoonotic transmission risk.

**Findings:**

The prevalence of *G. duodenalis* in humans and dogs was 18.3% (40/218) and 10.6% (10/94) by PCR, respectively. Molecular characterisation of the small subunit of ribosomal RNA (SSU rRNA) gene, triose phosphate isomerase (TPI) gene and sub-assemblage characterisation of the glutamate dehydrogenase (gdh) gene placed 27.5% (11/40) of *Giardia* positive humans into assemblage AII and 72.5% (29/40) into assemblage BIII of *G. duodenalis*. In dogs, 20.0% (2/10) of *Giardia*-positive samples were characterised as *G. duodenalis* assemblage BIII, 40.0% (4/10) as assemblage C and 40.0% (4/10) as mix infection between assemblage C and D.

**Conclusion:**

Overall, just over 2% of dogs harboured potentially zoonotic assemblages of *G. duodenalis* in the studied communities and hence pose a minimal zoonotic risk for the transmission of *Giardia* to humans.

## Background

*Giardia* is a protozoan flagellate that infects the intestinal tract of vertebrate hosts including humans and a considerable variety of other mammals through ingestion of infective cysts [1]. Among the six known *Giardia* species, only *Giardia duodenalis* has been found to infect humans and mammals. Eight distinct assemblages A to H have been reported and classified based on their genetic analysis and host specificity as reviewed by Ryan and Cacciò [2]. In detail, assemblages A and B can cause infection in humans and mammals, while assemblages C-G infect animals, but are occasionally found in humans. Moreover, assemblage C and D most commonly infect canids, assemblage E livestock, assemblage F cats, assemblage G rats and assemblage H marine mammals [2].

Dogs are predominantly infected with canid-specific genotypes of *G. duodenalis*, however may also harbour potentially zoonotic assemblages AI, AII, BIII and BIV [3]. This ability, combined with a high prevalence of infection in dogs [4] and their close association with humans, has made them a focus of attention as potentially zoonotic sources of *G. duodenalis.* In Cambodia, the prevalence of *Giardia* in humans varies from 2.9% to 8.0% [5–8]. In rural communities, semi-domesticated dogs share a close relationship with humans providing opportunity for the transmission of *G. duodenalis* to humans. Yet, documentation of prevalence and genotypes of *G. duodenalis* in canines and humans is virtually inexistent in many Southeast Asian countries and Cambodia in particular.

From a public health perspective, it is necessary to distinguish *G. duodenalis* cysts that primarily infect dogs (assemblages C and D) from those that have zoonotic potential (assemblages A and B), and this can only be based on their discrimination using a multilocus molecular typing approach [9].

In May 2012, a total of 40/218 (18.4%) of humans from 67 households in Dong village, Preah Vihear province, Cambodia were found positive for *Giardia* by PCR targeting the SSU rRNA gene of *G. duodenalis*. In dogs, the prevalence of *Giardia* using zinc sulphate centrifugal flotation was 2.1% (2/94) [10]. The objective of this study was to utilise more sensitive molecular tools to screen for the presence of *G. duodenalis* in dogs living in the same village and a multilocus genotyping approach to assess the risk for zoonotic transmission.

## Methods

### Study area, design and field procedures

For detailed information on study design and parasitological methods, refer to the study by Schär *et al*. [10]. Briefly, in May 2012, a total of 218 human and 94 dog faecal samples were collected from 67 households in Dong village, Rovieng district, Preah Vihear province, Cambodia (13.410842 N, 105.128217 E). This research was approved by the Ethics Committee of the Cantons of Basel-Stadt and Baselland, Switzerland, (EKBB, #18/12, dated 23 February 2012), and by the National Ethics Committee for Health Research, Ministry of Health, Cambodia (NECHR, #192, dated 19 November 2011). All studies with animals involved were done following the European Convention for the “Protection of Vertebrate Animals used for Experimental and Other Scientific Purposes”. Animals were handled with respect according to the laws on experimental animal care in Cambodia. All samples were transported at room temperature by TNT Express worldwide to the School of Veterinary Science, University of Queensland, Gatton campus, Australia for further analysis. All faecal samples were examined microscopically for the presence of *G. duodenalis* cysts using zinc sulphate centrifugal flotation. In addition, risk factors for infection of humans and animals were assessed by questionnaire interviews with participants and head of households.

### DNA extraction

Dog faecal samples fixed in 2.5% potassium dichromate were subjected to DNA extraction regardless of *Giardia* status by microscopy, using PowerSoil DNA Kit (Mo Bio, CA, USA). The extraction was performed according to manufacturer’s instructions with minor modification. ZIRCONIA/SILICA beads 0.5 mm in diameter (BioSpec product Inc, USA) were used instead of the PowerBead provided by the manufacturer. Samples were disrupted using a Mini-BeadBeater-16 (BioSpec product Inc, USA) at maximum speed for 5 min. The DNA was stored at -20°C until used for PCR amplification.

### Molecular analysis

#### The small subunit rDNA gene

A previously published nested PCR was utilised to screen dog faecal samples for *Giardia* by targeting a 174 bp fragment of the SSU rDNA gene of *G. duodenalis* [11, 12]. Briefly, the PCR was carried out in 25 μl volumes with each final reaction containing 1× CoralLoad PCR buffer (Qiagen Pty Ltd.), 12.5 pmol of each primer, 0.5 U of HotStar Taq DNA polymerase (Qiagen Pty Ltd.) and 2 μl of DNA. The cycling conditions were the same as the published protocol except for an initial denaturation of 5 min at 95°C. Dog and human [10] samples positive for *G. duodenalis* on the SSU rDNA gene were further characterised at the TPI gene.

#### The triose phosphate isomerase (TPI) gene

For humans, a pan-assemblage nested PCR targeting a 530 bp region of the TPI gene [13] and for dogs, an additional assemblage C- and D- specific PCR assay targeting a region of the TPI gene [14] was utilised to genotype *G. duodenalis* positive samples. The PCR was carried out in 25 μl volumes with each final reaction containing 1x CoralLoad PCR buffer (Qiagen Pty Ltd.), 12.5 pmol of each primer, 0.5 U of HotStar Taq DNA polymerase (Qiagen Pty Ltd.) and 2 μl of DNA. The cycling conditions were the same as the published protocol except for an initial denaturation of 5 min at 95°C. For all PCR assays, a positive control of *G. duodenalis* and a negative control of distilled water were included in each run.

#### The glutamate dehydrogenase (gdh) gene

Dogs and humans positive for *G. duodenalis* assemblage A and B at the SSU rDNA and TPI genes were further genotyped at a 530 bp region of the gdh gene using a previously published nested PCR assay [15]. The PCR cycling conditions were the same as the published protocol.

#### DNA sequencing

PCR positive samples of the correct size for the SSU rRNA, TPI and gdh genes of *G. duodenalis* were subjected to DNA sequencing. The PCR products were purified using PureLink quick PCR purification kit (Life Technologies, Invitrogen, Eugene, USA) according to manufacturer’s protocol and submitted to the University of Queensland Animal Genetic Laboratory for sequencing using both forward and reverse primers. DNA sequences were visualised using Finch TV 1.4.0 (Geospiza, Inc.) and aligned using BioEdit version 7.2.0 (http://www.mbio.ncsu.edu/BioEdit/bioedit.html) and directly compared with reference isolates of the TPI and gdh genes of *G. duodenalis* assemblages A-E.

#### Statistical analysis

The data was double entered and validated in Epidata (http://www.epidata.dk). Analysis was carried out using STATA version 12 (StataCorp ltd.). Infection prevalence rates were calculated. χ^2^ test was used to associate infection status with risk factors. A p-value <0.05 was considered significant.

## Results and discussion

A number of studies have demonstrated *G. duodenalis* as prevalent in both humans and dogs worldwide and have postulated the occurrence of anthroponotic, zoonotic and animal-specific cycles of transmission [9], which may be geographically and regionally unique in its epidemiology [16]. This study represents the first detailed report on zoonotic aspects of canine *Giardia* infections in Cambodia. The prevalence of *Giardia duodenalis* infection in human and dog samples by microscopic analysis and molecular analysis from Cambodia are displayed in Table 1. The prevalence among dogs of 2.1% by microscopy [10] and 10.6% by PCR is comparable to those encountered in rural communities in northeast India [17]. The primary difference between the two settings is that in Dong village, it would appear that the risk of dogs as a source of zoonotic *Giardia* is negligible. In dogs, 40.0% (4/10) of *G. duodenalis* positive samples were characterized as assemblage C, 40.0% (4/10) as mix infection between assemblage C and D and 20.0% (2/10) as assemblage BIII (Table 1). Overall, this placed 2/94 dogs (2.1%) as harbouring potentially zoonotic assemblages of *G. duodenalis* (Table 2). This is in contrast to other surveys in rural China, rural India, Spain and indigenous communities of Saskatchewan, in which assemblage A was the predominant zoonotic assemblage encountered in dogs [4, 18–20].

**Table 1:** **The prevalence of** ***Giardia duodenalis*** **infection in human and dog samples by microscopic analysis and molecular analysis from Cambodia**

Tests/species	Total no. of samples	Positive samples (%)	Assemblage A (%)	Assemblage B (%)	Assemblage C (%)	Assemblage D (%)
Microscopic						
Humans	218	20 (9.2)*	NA	NA	NA	NA
Dogs	94	2 (2.1)	NA	NA	NA	NA
PCR						
Humans	218	40 (18.4)*	11 (27.5)	29 (72.5)	-	-
Dogs	94	10 (10.6)	-	2 (20.0)	4 (40.0)	4 (40.0)

NA: not applicable, *Published by Schär *et al*. [10].

**Table 2:** **Summary of genotype results of** ***Giardia*** **isolates recovered from dogs at three different loci**

Dog isolate	SSU rRNA	TPI (broad specificity)	TPI (dog specificity)	gdh	Human isolate
H15D1	C	C	C	NA	
H17D1	C	C	C	NA	
H27D3	C	C	D	NA	
H28D7	C	C	D	NA	
H31D1	C	C	D	NA	
H32D1	C	C	C	NA	
H34D4	C	C	D	NA	
H36D1	B	B	B	BIII	H36H1
H37D1	B	B	B	BIII	
H37D3	C	C	C	NA	

NA: not applicable.

Among humans, the prevalence of *G. duodenalis* was 18.4% by PCR screening of the SSU rRNA gene, surpassing the microscopy-based prevalence of 9.2% described by Schär *et al*. [10]. Genetic characterisation placed 72.5% (29/40) of *G. duodenalis* as assemblage BIII and 11/40 (27.5%) as assemblage AII (Table 3). These results suggest that separate anthroponotic and dog-specific cycles of *G. duodenalis* exist within Dong village in Cambodia, with negligible potential for human-dog cross-transmission. Shared environments among dogs with indiscriminate defecation patterns are hypothesised to be conducive for the transmission of the canid-specific genotypes of the parasite [21, 22], as is the case for free roaming dogs in Dong village. Nevertheless, the opportunity for dogs to be exposed to human faeces within Dong village was still evident, reflected by just over half the human population utilising latrines [10]. This could have potentially accounted for the two dogs found to harbour *G. duodenalis* assemblage BIII genotypes, the predominant genotype in humans within the village. Whether these animals were indeed infected or just mechanically passing *Giardia* present in human faeces via coprophagy remains unknown.

**Table 3:** **Summary of genotype results of** ***Giardia*** **isolates recovered from humans at three different loci (A dash indicates unsuccessful PCR amplification or sequencing of the isolate)**

Human isolate	SSU rRNA	TPI	gdh
H03H1	B	B	BIII
H05H3	B	B	BIII
H05H4	B	B	BIII
H07H1	B	B	BIII
H16H1	B	B	BIII
H16H3	B	B	BIII
H16H4	B	B	BIII
H18H3	B	B	BIII
H20H4	B	B	BIII
H22H5	B	B	BIII
H25H3	B	B	BIII
H26H2	B	B	BIII
H27H1	B	B	BIII
H31H4	B	B	BIII
H35H1	B	B	BIII
H36H1	B	B	BIII
H41H1	B	B	BIII
DO112-02	B	B	BIII
DO124-03	B	B	BIII
DO124-06	B	B	BIII
DO126-01	B	B	BIII
DO126-03	B	B	BIII
D0130-02	B	B	BIII
D0132-05	B	B	BIII
DO134-03	B	B	BIII
DO135-03	B	B	BIII
DO140-03	B	B	BIII
DO141-03	B	B	BIII
DO144-02	B	B	BIII
H06H1	A	A	AII
H07H5	A	A	AII
H08H2	A	A	-
H17H4	A	A	AII
H22H3	A	A	AII
H40H4	A	A	AII
DO095-02	A	A	AII
DO119-04	A	A	AII
DO123-03	A	A	AII
DO140-04	A	A	AII
DO142-06	A	A	-

In this context it is important to note that multi-intestinal parasitic infections are highly frequent in Cambodia. In Dong village more than half of the village population harbours two or more intestinal parasitic infections concurrently. The same is true for dogs of the same households [10]. Interestingly, dogs in this village are known to contribute to the contamination of the environment with zoonotic parasites reflected by the highly prevalent nature of *Ancylostoma ceylanicum* in humans and dogs [23].

Age was the only factor that was significantly associated with the prevalence of *Giardia* in both humans [10] and dogs. The prevalence of *G. duodenalis* using PCR was significantly higher in young dogs up to 12 months of age (20.0%) compared to older dogs (3.7%, p = 0.02), a factor consistently observed in previous epidemiological studies [3, 21, 24]. Age increased immunity towards the parasite and risk-based exposures to infection are the most likely explanations for this observation.

This study confirms the advantage of utilising the assemblage C- and D-specific PCR [14] for targeting the TPI gene of *G. duodenalis* in dogs. Four out of 8 (50.0%) isolates from dogs classified as assemblage C using the 18S rRNA and the pan-assemblage PCR targeting the TPI [25] gene were classified as assemblage D using the assemblage C and D-specific assay (Table 3). As previously observed [9, 14], these are likely due to mismatches in the binding regions of the primers and most likely represent mixed assemblage C + D infections in these animals. However, the potential ability of *G. duodenalis* to undergo recombination between isolates from different assemblages may also account for this finding [26].

## Conclusion

This work contributes to the understanding of *G. duodenalis* transmission in human and dog populations living closely together in small rural communities in Cambodia and concludes that in rural Cambodia, community dogs play a negligible role as zoonotic reservoirs for *Giardia*.
